# Orthotopic Liver Transplantation for Budd-Chiari Syndrome: Observations from a 30-Year Liver Transplant Program

**DOI:** 10.3390/medicina57080821

**Published:** 2021-08-13

**Authors:** Marius Ibach, Dennis Eurich, Eva Dobrindt, Georg Lurje, Wenzel Schöning, Robert Öllinger, Johann Pratschke, Brigitta Globke

**Affiliations:** Department of Surgery, Campus Virchow-Klinikum and Campus Charité Mitte, Charité-Universitätsmedizin Berlin, Augustenburger Platz 1, 13353 Berlin, Germany; marius.ibach@charite.de (M.I.); dennis.eurich@charite.de (D.E.); eva-maria.dobrindt@charite.de (E.D.); georg.lurje@charite.de (G.L.); wenzel.schoening@charite.de (W.S.); robert.oellinger@charite.de (R.Ö.); johann.pratschke@charite.de (J.P.)

**Keywords:** liver transplantation, Budd-Chiari syndrome, anticoagulation, immunosuppression, clotting disorder, thrombophilia, hypercoagulable state

## Abstract

*Background and objectives* Budd-Chiari syndrome (BCS) refers to a complete thrombotic obstruction of the venous hepatic outflow tract due to various etiologies and constitutes a rare indication for ortothopic liver transplantation (LT). Few studies investigated long-term outcomes after LT for BCS. The aim of this study was to examine potential risk factors for late mortality and to evaluate long-term outcomes after LT for BCS. *Materials and methods:* 46 patients received an LT for BCS between 1989 and 2019 at the transplant center of the Charité-Universitätsmedizin Berlin. We analyzed potential effects of disease etiology, vascular events, rejection, and immunosuppression on long-term survival after transplantation using Kaplan-Meier curves and Cox logistic regression. *Results:* Of the 46 patients, 70% were female and 30% were male. Median age at the time of transplantation was 36 years. A total of 41 vascular events, including 26 thrombotic and 17 hemorrhagic incidents, occurred. The 1 year, the 5 year, the 10 year, and the 20 year survival rates were 87%, 83%, 76%, and 60%, respectively. By comparison, survival rates of the liver transplant cohort across all other indications at our center were slightly inferior with 85%, 75%, 65%, and 46%, respectively. In the study population, patients with myeloproliferative disorders showed worse outcomes compared to patients with other causes of BCS. *Conclusion:* Liver transplantation for BCS showed excellent results, even superior to those for other indications. Vascular events (i.e., thrombotic or hemorrhagic complications) did not have any prognostic value for overall mortality. Patients with myeloproliferative disorders seem to have a disadvantage in survival.

## 1. Introduction

During the 19th century, Rokitansky (1842) [1], Budd (1845) [2], and Chiari (1899) [3] provided the first descriptions of thrombotic or tumorous obstructions of the venous hepatic outflow—now commonly referred to as Budd-Chiari syndrome (BCS). Localization and extent of the obstruction can vary from the small hepatic veins to a complete thrombosis of the inferior vena cava [4]. Treatment options differ between subclinical and more severe manifestations and range from chronic anticoagulation to the necessity for a liver transplant. The age of manifestation is usually in young adulthood, with an incidence of 0.2–2 cases per 1 million [5]. Various innate or acquired thrombophilias may cause Budd-Chiari syndrome. Nearly half of the patient population suffers from a myeloproliferative disorder in which a Janus kinase 2 (JAK2) mutation can be detected in peripheral granulocytes or hyperplastic megakaryocytes in the bone marrow [6]. These Philadelphia-chromosome negative myeloproliferative syndromes (MPS) include essential thrombocytosis (ET), polycythemia vera (PV), and myelofibrosis and require different diagnostics and treatments compared to other causes of BCS, including cytoreductive therapy [7]. Other common causes comprise antiphospholipid syndrome, factor V Leiden thrombophilia, protein C and protein S deficiency, as well as paroxysmal nocturnal hemoglobinuria (PNH). While the use of contraceptives does not constitute an independent hazard, it can aggravate the risk associated with pre-existing clotting disorders [8]. Less frequently, tumor infiltration, autoimmune disorders, Dacarbazine therapy, trauma, or inferior vena cava webs may cause BCS. Untreated, the condition leads to end-stage liver failure and ultimately to death through progressive, hypertensive, and hypoxic destruction of hepatic tissue within approximately three years [9]. Diagnostics in acute and chronic situations include Doppler ultrasound (dUS), computer-assisted tomography (CT), or magnet-resonance imaging (MRI) of the hepatic veins and inferior vena cava. The underlying thrombophilia is diagnosed by lab results and bone marrow histology. Primary treatment consists of local and/or systemic therapeutic anticoagulation, prophylaxis of portal hypertension, radiological interventional therapies (i.e., angioplasty or transjugular intrahepatic portosystemic shunt procedure), and, in select cases, porto-caval shunting procedures. In cases of highly acute onset or if other treatments fail, patients require liver transplantation (LT) [10]. Besides treatment of the obstruction, the underlying hypercoagulable condition is usually treated with vitamin K antagonists [11]. Thus far, no clear and uniform recommendations for long-term management of liver transplant patients with BCS exist. In clinical practice, anticoagulation is based on the experience of the respective transplant center [12]. Earlier studies suggested that, while one year survival after liver transplantation for BCS was only 76%, life expectancy equaled that of routine indications at years five and ten [13]. To our knowledge, no long-term data with a follow-up of more than ten years in patients who underwent liver transplantation for BCS is yet published.

## 2. Materials and Methods

Long-term follow-up data on 46 BCS patients were obtained from the data base of the transplant clinic of the Charité-Universitätsmedizin Berlin, where all patients were followed-up with since 1989. The European data security protocol (DS-GVO) and the protocol for good clinical praxis of the Charité were followed. The inclusion criterion was liver transplantation due to BCS. Data were transferred from electronic and digitalized records and analyzed using SPSS (IBM SPSS Statistics for Windows, version 27, IBM Corp., Armonk, NY, USA) datasets. The variables analyzed were survival, vascular events (i.e. bleeding or thrombotic incidents), disease etiology, immunosuppression regimen, as well as demographic data (age, gender). The data collection was complete for all 46 patients. All patients who underwent LT at our center complete regular follow-up visits at our transplant outpatient clinic. The retrospective data collection and analysis were approved by the local ethics committee (EA1/035/21). Demographics are presented as mean and standard deviation of the mean. We also provide descriptive statistics on the etiology of BCS, treatment of the underlying conditions, and therapeutic management after LT including patient’s immunosuppression and anticoagulation. Postoperative vascular events (i.e. bleeding/thrombotic complications) and causes of death are presented as well as patient and graft survival of different sub-groups using Kaplan-Meier curves including the log-rank test statistics. Variables that appeared to affect mortality during Kaplan-Meier analysis were analyzed using a multivariate Cox regression analysis. 

## 3. Results

In total, 70% (*n* = 32) of patients transplanted for BCS were female and 30% (*n* = 14) male. The median age at the time of transplantation was 36 years (range 15–66) with a median follow-up of 20.8 years. A total of 22 (46%) patients suffered from myeloproliferative neoplasms, of whom nine (41%) were diagnosed with essential thrombocytopenia, five (23%) with polycythemia vera, and one (5%) with primary myelofibrosis. In seven (31%) patients, a Philadelphia-negative myeloproliferative syndrome with no further histological differentiation was diagnosed. Antibody mediated thrombophilias (*n* = 5, 11%) were subdivided into antiphospholipid syndrome in four patients and isolated heparin induced thrombocytopenia (HIT) in one patient. A total of eleven (24%) patients suffered from genetic thrombophilias: seven exhibited factor V Leiden mutations, three patients suffered from paroxysmal nocturnal hemoglobinuria, and one patient showed protein C deficiency (PCD); since PCD constitutes a hepatic synthesis defect, this patient was considered cured after transplantation and received no further anticoagulation. In all remaining cases (*n* = 8), the etiology of the thrombophilia was unknown. Eleven patients (24%) required re-transplantation. In five cases, the indication for re-transplantation was related to thrombotic events: three patients experienced recurrent BCS, and two patients suffered from hepatic artery thrombosis (Table 1). 

Eight acute rejection episodes occurred across the patient collective, of which six were initially resistant to steroids; we escalated therapy with Muronomab-CD3 in a single patient. In one case, a patient with chronic rejection required re-transplantation after 19 years. Two thirds of the patients received immunosuppression with calcineurin inhibitors (CNI). Antimetabolites were additionally used in one third of patients to reduce CNI exposure and associated toxicity. One patient with paroxysmal nocturnal hemoglobinuria received the monoclonal antibody Eculizumab, which inhibits C5-mediated terminal complement activation. We treated 95% of patients with concomitant vitamin K antagonists as primary anticoagulation with a target international normalized ratio between two and three, adding acetylsalicylic acid in less than 15% of cases. In total, 70% of patients with myeloproliferative neoplasms received cytoreductive treatment with anagrelide, hydroxyurea, or a combination of both. 

Despite adequate anticoagulation, 17 (37%) patients experienced 26 thrombotic incidents post-transplantation. In three patients, the outcome was fatal: one patient suffered from a myocardial infarction, and two died due to hepatic failure secondary to sinusoidal obstruction syndrome (SOS) and portal vein thrombosis, respectively. Non-fatal incidents comprised deep vein thrombosis (*n* = 6), portal vein thrombosis (*n* = 5), and stroke (*n* = 3). Therapy included increasing anticoagulation or radiological interventional lysis. In three patients with SOS, hepatic artery thrombosis, and portal vein thrombosis respectively, death occurred within 90 days after transplantation. All other thrombotic events (*n* = 23, 88%) were diagnosed in the long-time follow-up (Table 2).

Four patients suffered from recurrent BCS, including two patients with underlying myeloproliferative disease and paroxysmal nocturnal hemoglobinuria, respectively, of whom three required a re-transplantation. In two patients with PNH as the underlying disease, recurrence of BCS occurred during adjustment of anticoagulation due to an upper gastrointestinal (GI) hemorrhage in one and due to a minor planned surgery in the second patient. In one patient with isolated thrombosis of the hepatic veins, interventional lysis therapy was successful, but the patient suffered ischemic strokes years after this event despite adequate anticoagulation with vitamin K antagonists. The second patient required re-transplantation 13 months after the initial transplantation due to combined complete hepatic vein and portal vein thrombosis—however, no additional thrombotic or bleeding complications were documented for this patient during the further course.

In both patients with MPS as the underlying condition, recurrence of BCS developed under continuous vitamin K antagonist treatment. One patient required re-transplantation 8 months after initial transplantation and died due to a hemorrhagic stroke 25 days later. The second patient received a re-transplant 21 months after initial transplantation and died of biliary duct carcinoma of the transplant liver 21 years after re-transplantation with no further history of thrombotic or hemorrhagic events. Despite adequate anticoagulation, one patient with underlying myeloproliferative disease and one patient with protein C deficiency required re-transplantation secondary to hepatic artery thrombosis.

A total of 17 hemorrhagic events occurred in 15 (33%) patients (Table 3). 

Three patients died: one due to a spontaneous splenic rupture, a second due to postoperative bleeding, and a third patient due to intracerebral hemorrhage; all three received therapeutic anticoagulation at the time of the incident, and courses were fulminant, not allowing for alterations in anticoagulation therapy. The non-lethal events were primarily managed by tapering or temporary cessation of the therapeutic anticoagulation. Still, two patients required hysterectomies for severe hypermenorrhea. Five of these bleeding events (29%, i.e., postoperative hemorrhage, intracranial hemorrhage, GI bleeding, peri-hepatic hematoma, and one hemothorax) occurred within 90 days after transplantation. All other bleeding events occurred during long-time follow-up. 

**Figure 1:**
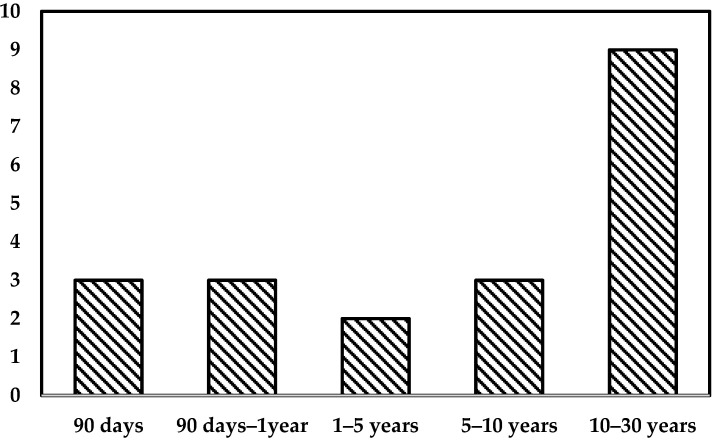
Time-dependent mortality post transplantation (*n* = 19, 41%).

The causes of death and the mortality distribution over time after transplantation for BCS are displayed in Table 4 and Figure 1. 

**Figure 2:**
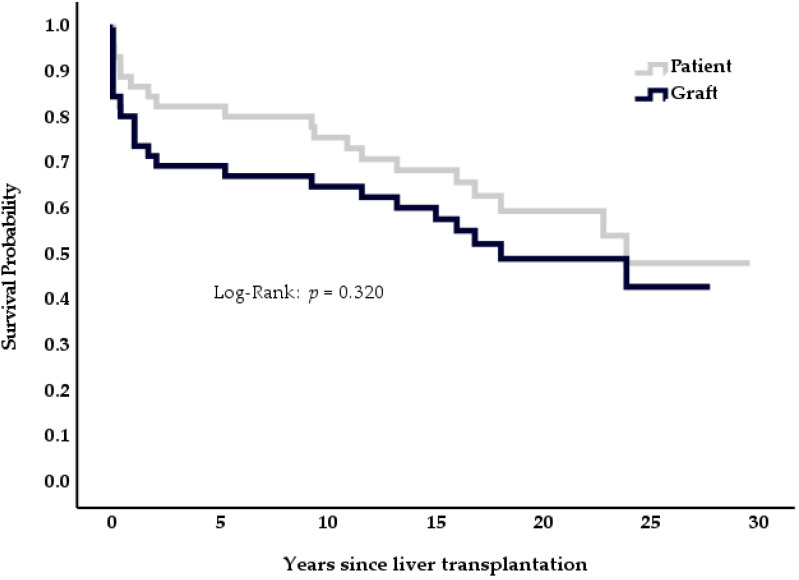
Cumulative graft and patient survival, patients with Budd Chiari syndrome (*n* = 46).

During the study period, 19 (41%) patients died between ten days and 24 years after transplantation. In three cases, death occurred within 90 days of transplantation: one patient suffered from multi-organ system failure secondary to sinusoidal obstruction syndrome ten days after the initial transplantation; two patients died within eleven and 22 days after transplantation due to multi organ failure and perioperative hemorrhage, respectively. Figure 2 displays patient and graft survival of the BCS cohort.

Graft losses occurred early after transplantation. Out of the eleven patients that needed a second transplant, only one was done 19 years after primary LT; all others were re-transplanted within the first three years after initial transplantation. 

**Figure 3:**
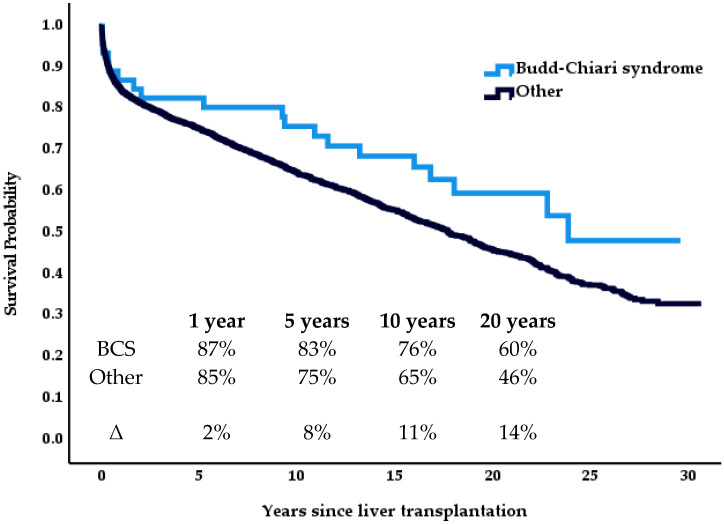
Cumulative survival—Budd-Chiari syndrome (*n* = 46) vs. other indications (*n* = 2677).

Between 1989 and 2019, 2723 patients received a primary liver transplant at our center. The median survival after LT for patients with BCS (*n* = 46) was 23.8 years, and 17.8 years for patients across all other indications (*n* = 2677). Cumulative survival rates at 10 and 20 years were 76% and 65% and 60% and 46%, respectively. This difference did not reach statistical significance (Figure 3, *p* = 0.103). 

**Figure 4:**
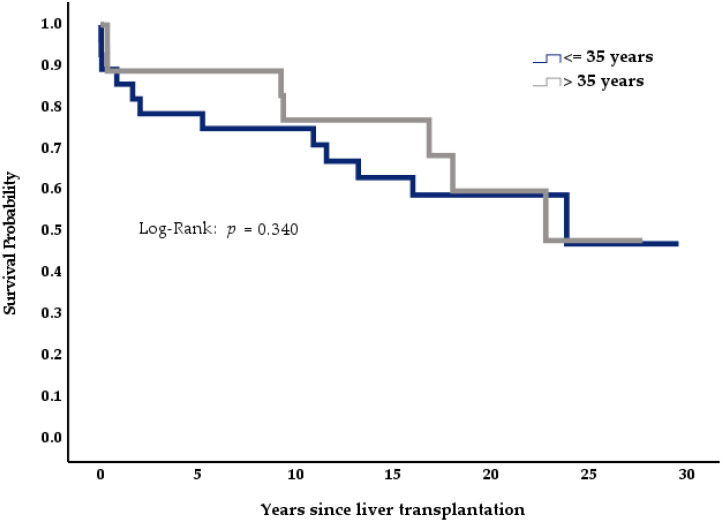
Impact of age on survival, *n* = 46.

**Figure 5:**
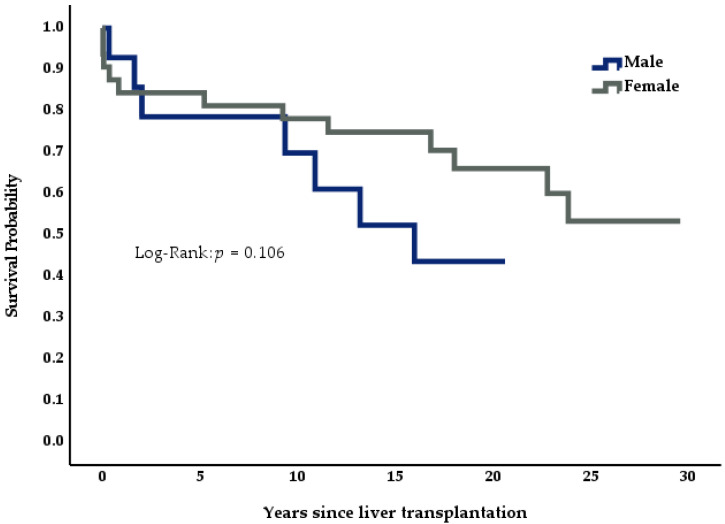
Impact of gender on survival, *n* = 46.

**Figure 6:**
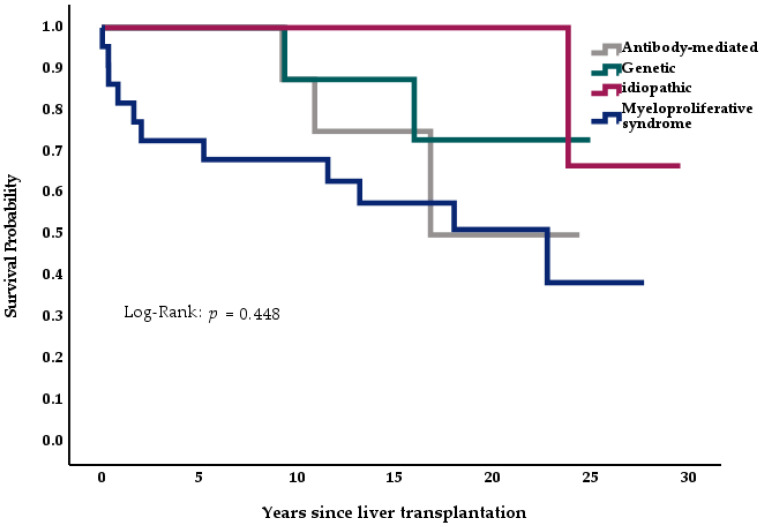
Impact of underlying disease on survival, *n* = 46.

We analyzed the effects of disease etiology and demographics on survival across the Budd-Chiari cohort (*n* = 46) using Kaplan-Meier curves, log-rank analysis, and multivariate Cox logistic regression. In the Kaplan-Meier curves, patients below the median age at the time of transplantation showed a tendency for better survival than older individuals (Figure 4, *p* = 0.340); female patients also showed better survival than their male counterparts (Figure 5, *p* = 0.106). Patients suffering from myeloproliferative disease seemed to have a higher mortality than patients with idiopathic BCS or genetic thrombophilias (Figure 6, *p* = 0.448).

We included variables that appeared to influence survival in the Kaplan-Meier analysis—age, gender, and disease etiology—in a multivariate Cox regression model (Table 5). 

This analysis revealed that patients with myeloproliferative disease (*p* = 0.037) and advanced age (*p* = 0.016) had significantly higher mortality rates compared to the rest of the BCS cohort. In a second model (Table 6), we added the occurrence of bleeding and thromboembolic events: neither variable influenced mortality (*p* = 0.954 and *p* = 0.745), while age and myeloproliferative disease remained significant (*p* = 0.021) and borderline significant (*p* = 0.054), respectively. 

Still, the only two variables with a significant effect on mortality remained age and the presence of a myeloproliferative syndrome (*p* = 0.010 and *p* = 0.041). 

Two individual cases demonstrate the potential difference in baseline risk in patients with underlying MPS. One patient with polycythemia vera died of septic shock secondary to progression to acute myelogenous leukemia 20 months after transplantation. In a second patient with myelofibrosis, splenic rupture occurred five years after transplantation. Pathologic examination revealed an acute leukemic process characterized by an increased blast count and significant extra medullary hematopoiesis in the spleen without signs of hepatic cirrhosis or portal hypertension.

## 4. Discussion

In this retrospective cohort study, we report long-term data on 46 patients who underwent liver transplantation for Budd-Chiari syndrome at the Charité Campus Virchow transplant center between 1989 and 2019. As a main result, Budd-Chiari patients showed a superior overall survival compared to the remaining collective over 30 years, exemplified by excellent 10 year and 20 year survival rates of 76% vs. 65% and 60% vs. 46%, respectively, with a median follow-up of 20.8 years.

Neither thrombotic nor bleeding events had statistically significant effects on survival. Our data show that, although bleeding (12%) and thrombotic events (29%) are a relevant cause for morbidity in the perioperative setting within 90 days after surgery, most of the events occur during long-term follow-up (88% of bleeding events and 71% of thrombotic events, respectively). This stresses the importance of continuous therapy with anticoagulants, as exemplified by four cases where cessation led to severe thromboembolic events: in one patient with underlying MPS, portal vein thrombosis led to fatal mesenteric ischemia two years after transplantation following cessation of vitamin K antagonists due to recurrent upper gastrointestinal bleeding episodes. This case resembled the course of two patients with underlying PNH, who suffered recurrent albeit non-lethal-BCS after discontinuation of the anticoagulation due to gastrointestinal hemorrhage and a small surgical procedure. Another patient suffered a myocardial infarction as anticoagulation was discontinued due to decreasing liver function. As cardiovascular and cerebrovascular events secondary to atherosclerotic changes are common causes of death in LT patients, the hypercoagulable state associated with BCS may aggravate the baseline risk in this patient group. 

Irrespective of the primary cause of the thrombophilia, caregivers of patients receiving an LT for BCS face the challenge of managing anticoagulation with a low risk of bleeding complications while establishing sufficient protection from thrombotic events. As of today, there are no uniform recommendations for long-term anticoagulation management in patients after LT and even less is established for patients transplanted for BCS. Traditionally, therapeutic anticoagulation was established with vitamin K antagonist. As the reasons for BCS are diverse, anticoagulation therapy could be adjusted according to hematological recommendations for the respective underlying condition if liver function is stable.

As an additional finding, visual trends in the Kaplan-Meier curves suggested that age, gender, and the underlying condition influence survival. Still, these results showed no statistical significance during log-rank analysis, likely due to the low patient number transplanted for this rare condition in our collective. However, when using a full model in a multivariate Cox regression analysis, both age and the presence of myeloproliferative disease significantly increased mortality. This observation remained significant even when controlling for gender, rejection, differences in immunosuppression, and vascular events. 

To increase sensitivity for more subtle effects, a collaboration between transplant-centers could raise the number of cases. Thus far, few studies addressed this topic. Mentha et al. [13] gathered data of 248 patients from 51 centers with a shorter follow-up of 10 years. In their sample, the distribution between the sexes was similar with around twice as many women receiving a transplant for Budd-Chiari syndrome than men; short- and medium-term outcomes (1–10 years) were comparable to those of the general transplant population. Their observations differed from the findings presented in this study: survival rates of our collective at the end of year 1 (87% vs 76%), year 5 (83% vs. 72%), and year 10 (86% vs 76%) exceeded those presented by Mentha et al. (2006) despite a cohort similar in age (36 years vs. 36 years). This also raises the question of whether the 14% gap in 20 year survival between BCS patients and more common indications at our center may solely be explained by a difference in age before transplantation (36 years vs. 49 years). 

One reason for this gap may be the excellent survival of patients with idiopathic disease in our sample. Patients in this cohort were, on average, 9 years older than the rest of the BCS population (44 years vs. 35 years) and only 5 years younger than the entire liver transplant cohort but showed vastly superior survival rates; all seven survived for more than 20 years after transplantation. Remarkably, we could not show that vascular incidents due to anticoagulation therapy in the BCS group led to a higher baseline risk for early mortality. One possible explanation is that cardiovascular events are one of the leading causes of death after liver transplantation [14] and in the general population [15], and chronic anticoagulation might prevent their occurrence—offsetting the increased risk for lethal bleeding incidents. To address the topic of groups at risk among the Budd-Chiari patients, further analysis of the underlying diseases is required. The different outcome of patients with myeloproliferative disease compared to patients with antibody-mediated or genetic thrombophilias might be based upon side effects of cytoreductive medication (e.g., leukopenia), a higher risk for secondary hematological neoplasia (e.g., acute myeloid leukemia (AML)) [16], or a disproportionate hypercoagulability. Patients with polycythemia vera are known to develop myelofibrosis in up to 15% of cases after 10 years and up to 50% after 30 years [17], and around 20% of patients develop AML [18]. These effects might be accelerated under immunosuppression, as exemplified by the described cases of patients with MPS, of whom one died of AML associated sepsis and the other due to a splenic rupture caused by a blast excess in the spleen. As Budd-Chiari constitutes a rare condition with multiple underlying causes, a more detailed analysis may be obtained through collaboration between transplant registries.

## 5. Conclusions

In summary, our data suggest that liver transplantation is an effective, life-saving measure with excellent long-term outcome in patients with terminal liver disease due to Budd-Chiari syndrome. While thrombotic and bleeding events did not influence overall survival, patients with myeloproliferative disease may have an increased risk for early death.

## Figures and Tables

**Table 1:** Descriptive characteristics of the study group: patients transplanted for Budd-Chiari syndrome (BCS), mean ± SD or *n* (%).

Patient Characteristics	
*n*	46
Female	32 (70%)
Male	14 (30%)
Age at transplantation, years	37 ± 11
Median (range)	36 (15–66)
**Underlying disease**	
Myeloproliferative syndrome	22 (48%)
Genetic thrombophilia	11 (24%)
Anti-body mediated thrombophilia	5 (11%)
Not determined	8 (17%)
**Known mutations**	
Factor V Leiden	7 (15%)
Janus Kinase 2	5 (11%)
Protein C deficiency	1 (2%)
**Anti-body mediated thrombophilia**	
Antiphospholipid syndrome	4 (9%)
Heparin induced thrombopenia	1 (2%)
**Cytoreductive therapy in MPS** * **patients (*n* = 22) post LT ^¥^**	
Hydroxycarbamide	12 (55%)
Anagrelide	3 (14%)
Hydroxycarbamide + anagrelide	2 (9%)
No cytoreductive therapy	5 (23%)
**Post-transplant anti-coagulation**	
Anti-vitamin K	35 (76%)
Anti-vitamin K + acetylsalicylic acid	5 (11%)
No anticoagulation therapy	2 (4%)
Not available	4 (9%)
**Immunosuppression**	
Calcineurin inhibitor	27 (59%)
Calcineurin inhibitor + antimetabolite	16 (35%)
Calcineurin inhibitor + prednisone	1 (2%)
Antimetabolite	1 (2%)
Not available	1 (2%)
**Re-transplantation**	11 (24%)
Initial non-functioning	4 (9%)
Recurrent Budd Chiari syndrome	3 (7%)
Arteria hepatica occlusion	2 (4%)
Chronic rejection	1 (2%)
Sinusoidal obstruction syndrome (SOS)	1 (2%)
**Rejection episodes**	9

* MPS = myeloproliferative syndrome, ^¥^ LT = liver transplantation.

**Table 2:** Venous and arterial occlusion events post transplantation, *n* (%).

Total Events	26
**Lethal**	
Sinusoidal obstruction syndrome	1 (4%)
Myocardial infarction	1 (4%)
Portal vein thrombosis	1 (4%)
**Non-lethal**	
Deep vein thrombosis	6 (24%)
Portal vein thrombosis	5 (20%)
Recurrent Budd Chiari syndrome *	4 (16%)
Stroke	3 (12%)
Hepatic artery occlusion *	2 (8%)
Myocardial infarction	1 (4%)
Mesenteric thrombosis	1 (4%)
Vena cava thrombosis	1 (4%)

* Three and two patients required re-transplantation in each group, respectively.

**Table 3:** Bleeding events post transplantation, *n* (%).

Total Events	17
**Lethal**	
Intracranial hemorrhage	1 (6%)
Splenic rupturePostoperative hemorrhage	1 (6%)1 (6%)
**Non-lethal**	
Hypermenorrhea *	4 (23%)
Gastrointestinal bleeding	5 (29%)
Hemothorax	2 (12%)
Ecchymosis	1 (6%)
Hemorrhagic renal infarction	1 (6%)
Peri-hepatic hematoma	1 (6%)

* Two patients required hysterectomy due to hypermenorrhea.

**Table 4:** Cause of death after liver transplantation for Budd-Chiari syndrome, *n* (%).

Total Deaths	19 (41%)
Multi organ system failure	4 (9%)
Transplant rejection	2 (4%)
Acute myelogenous leukemia	1 (2%)
Acute myocardial infarction	1 (2%)
Brain tumor	1 (2%)
Cholangiocarcinoma	1 (2%)
Cytomegalovirus pneumonia	1 (2%)
Heart failure	1 (2%)
Intracranial hemorrhage	1 (2%)
Osteosarcoma	1 (2%)
Perioperative hemorrhage	1 (2%)
Portal vein thrombosis	1 (2%)
Splenic rupture	1 (2%)
Sinusoidal obstruction syndrome	1 (2%)
Unknown	1 (2%)

**Table 5:** Multivariate Cox regression analysis of mortality: model A. Patients with BCS, *n* = 46.

Variable	Standard Error	Coefficient	*p*-Value	Relative Risk
Myeloproliferative disease	0.619	1.29	0.037 *	3.63
Gender (male)	0.025	0.59	0.206	2.07
Age (continuous)	0.594	0.25	0.016 *	1.07

* significant at α = 0.05.

**Table 6:** Multivariate Cox regression analysis of mortality: model B. Patients with BCS, *n* = 46.

Variable	Standard Error	Coefficient	*p*-Value	Relative Risk
Myeloproliferative disease	0.64	0.12	0.054	3.44
Gender (male)	0.63	0.75	0.231	2.13
Age (continuous)	0.03	0.06	0.021 *	1.07
Bleeding	0.63	0.04	0.954	1.04
Thrombosis	0.62	−0.20	0.745	0.82

* significant at α = 0.05.In the third model (Table 7), we included the use of mycophenolate mofetil—which can reduce total blood cell count—and rejection episodes as additional factors.

**Table 7:** Multivariate Cox regression analysis of mortality: model C. Patients with BCS, *n* = 46.

Variable	Standard Error	Coefficient	*p*-Value	Relative Risk
Myeloproliferative disease	0.70	1.38	0.048 *	3.99
Gender (male)	0.70	0.74	0.290	2.09
Age (continuous)	0.03	0.70	0.023 *	1.07
Additional MMF ^a^	0.64	−0.07	0.920	0.94
Rejection episode	0.70	0.49	0.484	1.63

* significant at α = 0.05; ^a^ Mycophenolate mofetil.

## Data Availability

Retrospective data was collected from the Charité internal transplant database (not publicly available).
